# Length of stay after childbirth in India: a comparative study of public and private health institutions

**DOI:** 10.1186/s12884-020-2839-9

**Published:** 2020-03-23

**Authors:** Pradeep Kumar, Preeti Dhillon

**Affiliations:** 1 International Institute for Population Sciences, Mumbai, Maharashtra 400088 India; 2 Department of Mathematical Demography and Statistics, International Institute for Population Sciences, Mumbai, India

**Keywords:** Postnatal care, Discharge from the hospital, Length of stay, Public, Private, Vaginal, Cesarean delivery

## Abstract

**Background:**

This paper discusses length of stay (LOS) following childbirth as an indicator of quality of postnatal care in health institutions. This research aims to describe LOS according to both vaginal and cesarean deliveries in public and private health care institutions in India, and to identify any association of LOS with postnatal care and post-delivery complications.

**Methods:**

We use recently released nationally-representative data from the National Family Health Survey-4 (2015–16) and apply the Cox proportional hazard model to determine the factors associated with LOS at the health facility after childbirth during a five-year period preceding the survey.

**Results:**

Overall, the average LOS after childbirth is 3.4 days; 2.1 days for vaginal deliveries and 8.6 days for cesarean section (CS) deliveries. Strikingly, half of the women are discharged within 48 h. Women who give birth in private hospitals have a more prolonged stay than those who give birth in public health facilities. For vaginal birth in public hospitals, one-fourth of the women are discharged with insufficient LOS as against only 19.2% women in private hospitals. LOS is significantly related to the cost of delivery only in the case of private facilities. Uneducated women belonging to lower wealth quintile households and those living in rural areas stay for a shorter duration for vaginal deliveries but for a longer duration in case of cesarean deliveries. Women who get four or more antenatal check-ups (ANC) done have a longer stay, while those who receive benefits under the *Janani Suraksha Yojna* (JSY) have a shorter stay. Another key finding is that women who are discharged on the same day report lower levels of postnatal care and a higher proportion of post-delivery complications.

**Conclusion:**

The study concludes that early discharge has a negative association with maternal health outcomes, which has important program implications. Therefore, it is essential to maintain an adequate LOS at a facility after childbirth. We recommend that government programs should strengthen the JSY scheme not only to improve delivery care, but also to provide effective postnatal care by promoting sufficient LOS at facilities.

## Background

Global efforts to prevent maternal and perinatal mortality aim to ensure all women have access to skilled attendants for childbirth, which in practice is synonymous with advocating for both facility delivery and home birth. In the twentieth century, a higher proportion of births occurred in a facility/hospital than at home [1–3]. The maternal mortality ratio (MMR) in India had declined by 124 points from 254 maternal deaths per 100,000 live births in 2006 to 130 in 2016 [4]. There has been a surge in institutional births, which has led to a reduction in the maternal mortality ratio [5, 6]. The implementation of government of India initiatives, like the National Rural Health Mission (NRHM) and the Janani Suraksha Yojna (JSY) program, from early 2005 onwards has shown a considerable impact on maternal health care utilization in India as a whole. The JSY program encourages institutional births by providing cash incentives to pregnant women and Accredited Social Health Activists (ASHAs). This has led to a reduction in the MMR, especially in states with a high maternal mortality. However, the dramatic increase in institution-based deliveries has not resulted in the anticipated drop in maternal complications. It is important to determine why so that limited resources can be used in more effective ways possible. Literature indicates that JSY has made a difference in terms of institutional births [7]. Evidence suggests that the implementation of JSY may result in an increase in institutional births, but that it may not decrease the MMR to the same extent [8]. Length of stay after childbirth is also an important factor influencing MMR, but it has not increased to the same degree as institutional birth. There was a gigantic increase in institutional births from 39% in 2005–06 to 79% in 2015–16. During the same period, the percentage of mothers who received a post-natal check-up in the first 2 days after birth increased from 37% to 65% only [9], and maternal complications increased as well [9].

Half of all post-delivery maternal deaths occur during the first week after childbirth, and a very high proportion of these deaths occurs during the first 24 h after delivery [10]. The institutional birth strategy stems from the recognition that most potentially fatal complications cannot be predicted and that having professionally trained midwifery care is indispensable to handle such complications when they arise. According to the WHO (2015), the first 24 h postpartum is the highest risk period for women and newborns [11, 12]. For both the mother and the infant, immediate postnatal care is important to treat complications, like postpartum infections, excessive bleeding, pain in the perineal area, vaginal discharge, etc., that arise from birth and to provide the mother with relevant information on caring for herself and her baby. The extent to which women receive such care is directly related to the length of their postpartum stay. Postpartum length of stay after vaginal delivery has been identified as an essential quality indicator of woman care [2, 13, 14]. The length of stay after cesarean delivery may also be important for evaluating the quality of peripartum and postpartum obstetric care.

Length of hospital stay is likely to be longer after a CS (an average of 3–4 days) than after a vaginal birth (average 1–2 days). But women who are recovering well, are apyrexial, and do not have complications following CS should be offered early discharge (after 24 h) from hospital and follow-up at home because this is not associated with more infant or maternal readmissions [15]. However, other studies have found that short lengths of stay may leave insufficient time to detect, diagnose, or treat complications, which can, in turn, increase morbidity and mortality [12, 16, 17]. Study evidence shows the four most prevalent health problems, viz. fatigue, insomnia, breast problems and constipation, among Turkish women after early discharge [18]. Women who are discharged early after childbirth are significantly more likely to be depressed than those who stay in the hospital for a longer period [19–21]. Another study shows that women with an early discharge (one night) from the hospital report fatigue more frequently, worry more about their baby’s health, and report higher neonatal morbidities [22]. Hence, previous research suggests that early discharge after childbirth has a negative impact on women’s health. In contrast to this, a few studies show that early postnatal discharge of healthy mothers and term infants does not appear to have any adverse effects when the discharge is accompanied by a policy of offering women at least one nurse-midwife home visit post-discharge [23].

The previous research has reported the average length of stay after childbirth in a health facility to be between 1.3 and 6.6 days for vaginal deliveries and between 1.4 and 9.3 days for cesarean deliveries, as shown in the following table that presents reviewed literature on the average length of stay.

**Table:** 

Study	The average length of stay reported by various studies		Study area
	Vaginal delivery	Cesarean delivery	
Campbell et al. 2016 [12]	1.3–6.6 days	2.5–9.3 days	92 countries
Acharya et al. 2016 [24]	4 days	7 days	Nepal
Mamun et al. 2011 [25]	4 days	6.21 days	Brisbane, Australia
Rice et al. 1999 [26]	Less than 4 days	6 and more days	Victoria, Australia
Wen et al. 1998 [27]	1.5 days	1.4 days	Canada
Leung et al. 1998 [28]	1.18 days	2.71 days	California
Ford et al. 2012 [29]	–	6 and more days	New South Wales

A study by Liu et al. [30] in Canada reported that 13% of the women stayed at the facility for less than 24 h after a vaginal delivery [30]. Women having complications before, during, or after childbirth stay longer after birth [30–32]. Healthy mothers, on the other hand, have the shortest postpartum hospital stays. Maternal asthma and gestational weight gain influence maternal stay after delivery [25, 33]. Women with perioperative complications are at a higher risk of an extended postpartum length of stay. A study based on the impact of postpartum haemorrhage (PPH) on hospital length of stay reported that women with postpartum haemorrhage experienced a significantly longer length of stay and had higher inpatient mortality rates than women without postpartum haemorrhage [34].

Postpartum length of stay may depend on several factors like type of facility, mode of delivery, obstetric morbidity, and socioeconomic and demographic characteristics of the women. Earlier research has found that women from the older age group, those of higher socioeconomic status and education, and those having a private payor source have the most extended length of stay. In contrast, early discharge is associated with young age, multi-parity, low socioeconomic status, and lack of readiness for discharge [19, 35]. Length of stay after childbirth is steadily declining in the UK and other countries also due to cost saving. The growing cost of hospital stays is one of the factors for early discharge [36]. In addition to this, Blumenfeld found that younger and older women (less than 20 years or more than 34 years of age) who have insurance are more likely to stay for long after birth [2].

It is clear from the above literature review that post-delivery stay in health facilities is not well documented, particularly in India. A limited research, which emphasizes the length of stay and type of care given to women and children after delivery in a health facility, is available. Therefore, the present study is an attempt to fill this research gap to describe LOS according to both vaginal and cesarean deliveries in public and private health care institutions in India, and to identify any association of LOS with postnatal care and post-delivery complications.

### Data and methodology

The study is cross-sectional in nature and based on secondary data from the fourth round of the nationally-representative survey National Family Health Survey (NFHS) conducted in 2015–16. Data can be obtained on request from the International Institute for Population Sciences, Mumbai, India or from the Demographic and Health Survey (DHS). NFHS-4 provided information on population, health, nutrition, abortion, sexual behaviour, HIV/AIDS knowledge, attitudes, behaviour, and domestic violence for India as well as each state, union territory, and district of India. NFHS used the stratified multi-stage cluster random sampling procedure for the selection of the sample and covered 29 states, seven union territories, and 640 districts. More details on the survey design and sample size can be found in the national report [9]. The study collected data from 148,185 women, aged 15–49, about their last institutional delivery conducted during 5 years preceding the survey. A certain specific set of questions were asked using standard questionnaires with the consent of the respondents. These women were asked, “How long after delivered did you stay in the health facility?” Responses were recorded in hours for those who stayed less than 24 h; in days for who stayed for less than 7 days; and in weeks for those who stayed more than a week. We transformed these responses into number of days for calculating the average length of stay. Four per cent of the women reported more than 7 days of stay, and 0.31% reported not knowing the length of stay. These small proportions of women are unlikely to have affected the estimates of the mean length of stay. We estimated the mean length of stay after childbirth separately for cesarean and vaginal deliveries and also for public and private hospitals. The study used two-way ANOVA for testing the mean length of stay between groups that were split on two independent variables. According to the World Health Organization, the first 24 h are critical for both mother and newborn. Also, postnatal care within 48 h after childbirth is a good indicator for women’s health. Within this time period, we can assume that women get sufficient time to receive essential postnatal care services. Taking this into consideration, the present study categorized the continuous variable of LOS into three categories for vaginal births: insufficient stay (less than 24 h), sufficient stay (24–47 h), and extended stay (more than 48 h).

We estimated the average length of stay by background variables of women and by mode of delivery. The study applied the Cox proportional hazard model to determine the factors affecting the risk of discharge after delivery. Here, time is defined as the “length of stay,” and the failure event is “woman’s discharge” from the facility. Therefore, time-to-event varies across individuals, and censoring of events exists. The form of the Cox proportional hazard model used is given below:

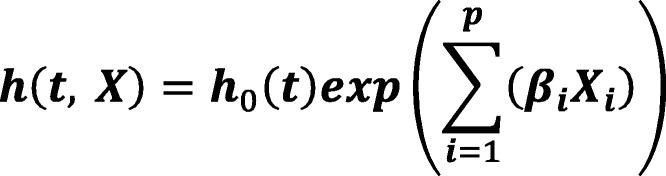



where X = (X_1_, X_2_, … … ..X_p_) are the explanatory/predictor variables, and h_0_(t) is called the baseline hazard.

Here, the hazard ratio represents the coefficient value, that is, the ratio of a woman’s discharge from the facility at a given time. Predictor variables include age of women (15–24, 25–29, 30–39 and 40+ years), place of residence (Rural and Urban), education of the women (Illiterate, Primary, Secondary and Higher). Caste grouped into four categories; Scheduled Caste, Scheduled Tribe, Other Backward Class, and Others (including all privileged caste groups). Among the four categories of castes, people belonging to Schedule caste lies at the bottom of the Indian caste system and have been exploited for over centuries. The indigenous groups of India belong to the category of Schedule tribes and are among the most deprived sections [37]. The one falling under the category of Other Backward Class are socially and educationally backward. However, they are distinct from Schedule Caste and Schedule tribes [38]. Other predictors are religion (Hindu, Muslim, and Others,), wealth index of households (Poorest, Poorer, Middle, Richer, and Richest), parity (1st, 2nd, 3rd, and 4th+), number of antenatal checkup visits (ANC) (No visit, 1–3 visits, 4 and more visits), Janani Suraksha Yojana (JSY) assistance (Not received and received), mode of delivery (Vaginal and Cesarean), type of delivery (Public and Private), and complications during delivery (if a woman had breech presentation or prolonged labour or excessive bleeding; anyone among them). Information on these predictors was directly sought in the survey. The wealth index, however, was derived from the survey based on questions related to household amenities and conditions.

Public facilities were taken to include government/municipality hospital, government dispensary, urban health clinic/urban health post (UHP)/urban family welfare center (UFWC), Community Health Centre (CHC)/Rural Hospital/Block Primary Health Care (BPHC), PHC/Additional PHC, Sub-Centre, and other public sector health facility, whereas private facilities were taken to include private hospital/maternity home/clinic, other private sector health facility, and NGO or trust hospital/clinic.

This paper also analyses the association of complications, including massive vaginal bleeding after childbirth/postpartum hemorrhage (PPH) and fever (in the first 2 months after delivery) with postnatal care within 48 h (Yes and No) and delivery cost. The survey sought information on fever (self-reported and not measured with a thermometer) in the following manner: “If a woman reports that she had very high fever, record it as ‘1’ irrespective of the cause of the fever so long as it was within the first two months postpartum.”

## Results

A total of 148,185 women reported having a live birth delivered in a facility during 5 years prior to the survey. The length of stay for vaginal and C-section deliveries by public and private health institutions is shown in Table 1. Overall, the average length of stay at any health facility is 3.4 days. It is 2.9 days in public facilities and 4.7 days in private health facilities. As expected, women who have vaginal deliveries stay for a shorter length (2.1 days) after childbirth; though, the length of stay does not vary much by public and private health facilities. On the other hand, women stay for a longer time (8.6 days) after having C-section deliveries. Interestingly, in the case of cesarean deliveries, women admitted in public facilities stay for a longer time than those admitted in private health facilities.

**Table 1:** Percent distribution of women by their length of stay (LoS) and average LoS after childbirth in public and private health facilities according to type of delivery, India, 2015-16

Duration of Stay (in days)	Total***			Vaginal***			C-section***		
	Public	Private	All	Public	Private	All	Public	Private	All
**0**	21.2	12.8	18.2	23.4	19.2	22.3	6.7	4.3	5.1
**1**	20.3	15.5	18.6	22.6	24.6	23.1	5.6	3.5	4.3
**2**	16.9	12.8	15.4	18.8	20.3	19.2	4.3	3.0	3.4
**3**	22.6	15.6	20.1	25.1	20.8	24.0	6.3	8.6	7.7
**4**	3.2	6.2	4.2	3.0	4.5	3.4	4.6	8.4	7.0
**5**	3.6	10.7	6.1	2.6	4.8	3.2	10.0	18.3	15.3
**6**	1.7	4.6	2.7	0.8	1.2	0.9	7.8	9.1	8.6
**7**	6.5	15.2	9.6	2.0	2.8	2.2	36.3	31.6	33.3
**Above 7**	4.0	6.8	5.0	1.8	1.8	1.8	18.5	13.3	15.2
**Average**	**2.9**	**4.7**	**3.4**	**2.1**	**2.2**	**2.1**	**8.9**	**8.5**	**8.6**
**Number of women**	**1,05,615**	**42,570**	**1,48,185**	**92,969**	**25,478**	**1,18,447**	**12,646**	**17,092**	**29,738**

*p* < 0.0001 *** based on chi-square test

The study found that a significant percentage of women are discharged early. Overall, around 36.8% (41.5% in public facilities and 28.3% in private facilities) of the women are discharged before completing 2 days of stay and 18% (21.2% in public facilities and 12.8% in private facilities) within a day. Early discharge is more common in the case of deliveries conducted in the public sector than in the private sector for both modes of deliveries. In the public sector, nearly 23.4% of the women with normal deliveries and 6.7% of the women with cesarean deliveries are discharged on the same day after childbirth. However, late discharge (after a week) is more common among women who have cesarean deliveries in public facilities (18.5%) as compared to those who have them in private facilities (13.3%).

**Fig. 1:**
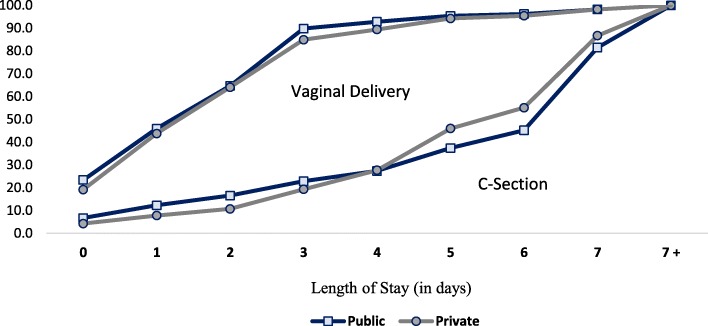
Cumulative percentage of women by their length of stay at the institution after delivery

The cumulative percentage of women by number of days after birth when they were discharged is shown in Fig. 1. It is clearly visible that in the case of vaginal delivery, women are discharged early and that there is only a minor difference between public and private sectors on this front. On the contrary, in the case of cesarean deliveries, early discharge is more common in public hospitals. This trend is seen until the fourth day after delivery, after which, a higher proportion of women are discharged from the private hospitals.

**Fig. 2:**
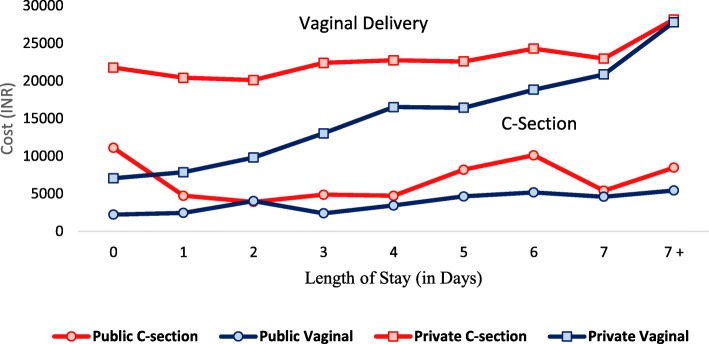
Average cost of delivery by length of stay

In this study, we also assess the link between the length of stay and the cost of delivery. The average delivery cost by number of day on which women are discharged after birth is given in Fig. 2. The figure reveals that the cost of delivery is higher in private facilities than in the public ones. With increase in the number of days of stay, the cost of delivery increases too, but this is true only in the case of private health facilities. The cost of delivery in private facilities increases sharply after 2 days of stay, and more so in the case of vaginal delivery. Obviously, the cost of cesarean delivery is higher than that of normal delivery. After the 7th day of stay, vaginal delivery cost meets with cesarean delivery cost.

The average length of stay of women after childbirth by their socioeconomic and demographic characteristics for both modes of deliveries and by health facilities is presented in Table 2. The length decreases with increase in the age of women irrespective of the mode and place of delivery. For vaginal deliveries, women with 3 and 4 or more parity are discharged earlier (in 1.91 and 1.66 days respectively) as compared to first parity women (2.34 days). This pattern is true for both places of delivery. For vaginal deliveries, length of stay of women after childbirth increases with their level of education, which is not true for cesarean deliveries. Higher educated women stay on an average for 2.6 days as compared to illiterate women (1.6 days) for vaginal births. There is a positive relationship between place of delivery and education of women, and the length of stay is longer in private facilities compared to public health facilities.

**Table 2:** Average length of stay (in days) of women after childbirth in public-private health facility by type of delivery according to their background characteristics, India, 2015-16

Duration of Stay (in days)	Total			Vaginal			C-section		
	Public	Private	All	Public	Private	All	Public	Private	All
**Age**			***			***			***
15–24 Years	2.90	4.69	3.36	2.15	2.08	2.14	9.26	9.12	9.18
25–29 Years	2.95	4.71	3.47	2.12	2.31	2.16	9.11	8.25	8.60
30–39 Years	2.88	4.81	3.49	1.99	2.35	2.08	8.34	8.10	8.20
> 40 Years	2.29	4.82	2.96	1.72	2.02	1.78	6.87	8.27	7.70
**Education**			***			***			***
Illiterate	2.13	3.86	2.42	1.75	1.56	1.72	8.19	9.68	8.92
Primary	2.68	4.35	3.00	2.07	1.86	2.04	9.20	9.31	9.25
Secondary	3.25	4.87	3.72	2.26	2.39	2.29	9.03	8.53	8.76
Higher	3.70	5.03	4.47	2.27	2.56	2.41	8.66	7.73	7.97
**Place of Residence**			***			***			***
Urban	3.62	4.68	4.07	2.48	2.42	2.46	8.57	7.64	7.98
Rural	2.69	4.78	3.17	1.98	2.13	2.01	9.05	9.14	9.10
**Religion**			***			***			***
Hindu	2.93	4.88	3.49	2.12	2.21	2.14	9.64	8.73	9.09
Muslim	2.81	4.19	3.24	1.90	2.10	1.95	7.14	7.76	7.44
Others	2.80	4.50	3.24	2.06	2.77	2.20	7.51	7.13	7.32
**Caste**			***			***			***
Schedule Castes	3.00	4.73	3.36	2.15	2.12	2.15	9.81	8.68	9.26
Schedule Tribes	2.67	4.55	2.94	2.16	2.59	2.20	8.02	8.03	8.02
Other backward class	2.84	4.82	3.50	2.02	2.20	2.07	9.84	9.02	9.31
Others	3.06	4.58	3.67	2.08	2.20	2.11	8.18	7.73	7.89
**Wealth quintile**			***			***			***
Poorest	2.04	4.19	2.28	1.72	1.65	1.71	9.02	10.41	9.64
Poorer	2.63	4.68	2.95	2.06	1.87	2.03	8.90	10.61	9.62
Middle	3.34	5.02	3.75	2.28	2.23	2.27	9.85	9.48	9.68
Richer	3.56	5.04	4.11	2.37	2.42	2.39	8.83	8.64	8.72
Richest	3.39	4.50	4.02	2.22	2.42	2.32	7.60	7.11	7.24
**Parity**			***			***			***
1st Parity	3.37	5.11	3.96	2.30	2.47	2.34	8.91	8.27	8.53
2nd Parity	3.14	4.91	3.67	2.19	2.30	2.21	9.29	8.60	8.90
3rd Parity	2.34	3.99	2.73	1.90	1.95	1.91	7.76	8.50	8.18
More Parity	1.89	3.22	2.14	1.66	1.68	1.66	7.81	9.07	8.52
**Any complication during delivery**			***			***			***
No	2.83	4.56	3.32	2.07	2.20	2.10	8.96	8.34	8.60
Yes	3.00	4.96	3.57	2.11	2.30	2.15	8.79	8.57	8.66
**Number of ANC**			***			***			***
No visits	2.24	4.41	2.69	1.72	2.13	1.79	10.05	9.75	9.89
1–3 visits	2.31	4.24	2.75	1.77	1.81	1.78	8.93	9.10	9.03
4 & more visits	3.51	4.98	4.02	2.46	2.48	2.46	8.74	8.13	8.38
**Received JSY Assistance**			***			***			***
No	3.44	4.71	4.05	2.38	2.23	2.32	9.12	8.37	8.60
Yes	2.51	5.09	2.61	1.90	2.50	1.91	8.58	9.85	8.75
**N**	**1,05,615**	**42,570**	**1,48,185**	**92,969**	**25,478**	**1,18,447**	**12,646**	**17,092**	**29,738**

*p* < 0.0001 *** based on two-way ANOVA

The average stay among urban women is longer (4.07 vs 3.17) compared to their rural counterparts for vaginal deliveries, while the trend goes in the other direction (7.98 vs 9.10) for cesarean deliveries. The mean length of stay among Muslim women is 3.24 days, and it is higher in private (4.19) than in public facilities (2.81). Overall, scheduled tribe women are discharged earlier (2.94) than women of all other caste groups. Caste doesn’t play a significant role in determining the length of stay for vaginal deliveries. However, for cesarean deliveries, the length of stay is lower in women of general caste than among those belonging to lower socio-economic groups (SCs, STs) and OBCs. For vaginal deliveries, women from the poorest households are discharged in about 1.7 days in contrast to those from the affluent households who are discharged in about 2.3 days irrespective of the place of delivery. However, in the case of cesarean deliveries, women from the richest households are discharged sooner (7.24 days) as compared to women from the poorest families (9.6 days).

Women who experience some kind of delivery complication stay longer at facilities irrespective of the mode and place of delivery. The number of ANC visits has a significant role in women’s length of stay in a health institution, particularly in a public health facility. Women who give birth in a public health facility and make four and more ANC visits stay for 2.46 days for vaginal deliveries and for 8.74 days for cesarean deliveries. Women who do not go for any ANC, on the other hand, stay for 1.72 days for vaginal deliveries and 10.05 days for cesarean deliveries. In addition, women who receive the JSY assistance for delivery in a public health facility stay for a shorter length than who do not get this assistance.

Insufficient, sufficient and extended LOS among women after vaginal birth by type of facility according to their background characteristics is presented in Table 3. Results show that one-fourth of the women are discharged with insufficient LOS for vaginal birth in public hospitals in contrast to only 19.2% of the women in private hospitals. However, the percentage of women with sufficient LOS is more in private (24.4%) facilities than in public (22.4%) health facilities. The proportion of women discharged with insufficient LOS is higher in public than in private health facilities irrespective of the age of women. For vaginal births, about one-third of illiterate women are discharged with insufficient LOS irrespective of the place of delivery. Twenty per cent of higher educated women are discharged with sufficient LOS in both public and private hospitals. In public hospitals, 13.6% women from urban and 26.4% women from rural areas are discharged with insufficient LOS. However, in private hospitals, 22% women from urban and 26.2% from rural areas are discharged with sufficient LOS. Interestingly, in public hospitals, 28% OBC women are discharged with insufficient LOS as against 21% in private hospitals. As far as women from other castes are concerned, around one-fourth of them are discharged with sufficient LOS irrespective of the place of delivery. Among the poorest women, one-third are discharged with insufficient LOS in both public and private hospitals. As many as 21.3% women with any delivery complication in public hospitals are discharged with insufficient LOS, whereas this figure is only 17% in private hospitals. Surprisingly, 27% women who receive the JSY assistance are discharged with insufficient LOS in public hospitals, though this figure is only 17.3% in private hospitals.

**Table 3:** Insufficient, sufficient, and extended LOS among women after vaginal births by type of facility according to their background characteristics, India, 2015–16

Background characteristics	Public			Private		
	Insufficient	Sufficient	Extended	Insufficient	Sufficient	Extended
Age	*P* < 0.000			*P* < 0.000		
15–24 Years	21.4	21.2	57.4	19.7	25.8	54.6
25–29 Years	23.1	22.2	54.7	17.9	23.7	58.4
30–39 Years	26.3	24.3	49.5	20.3	23.6	56.1
> 40 Years	35.0	28.0	36.9	24.6	26.8	48.6
Education	*P* < 0.000			*P* < 0.000		
Illiterate	32.1	25.4	42.6	32.7	31.5	35.8
Primary	22.8	23.5	53.7	23.5	28.5	48.0
Secondary	18.9	20.6	60.5	17.2	23.9	58.9
Higher	20.4	20.1	59.6	13.4	19.3	67.4
Place of Residence	*P* < 0.000			*P* < 0.000		
Urban	13.6	18.0	68.4	14.2	22.0	63.8
Rural	26.4	23.7	49.9	23.1	26.2	50.7
Religion	*P* < 0.000			*P* < 0.000		
Hindu	23.5	22.1	54.4	19.3	24.9	55.8
Muslim	26.0	23.2	50.8	21.8	22.5	55.8
Others	13.4	25.1	61.5	10.8	22.7	66.5
Caste	*P* < 0.000			*P* < 0.000		
Schedule Castes	22.5	22.5	54.9	22.3	25.3	52.4
Schedule Tribes	16.4	21.4	62.2	15.0	27.7	57.3
Other backward class	28.0	21.9	50.1	21.3	24.3	54.4
Others	18.9	23.8	57.3	15.3	23.6	61.1
Wealth quintile	*P* < 0.000			*P* < 0.000		
Poorest	33.4	26.1	40.5	34.3	28.1	37.6
Poorer	24.3	23.4	52.3	28.4	29.6	42.0
Middle	19.6	20.5	60.0	21.0	25.1	53.9
Richer	16.1	18.7	65.3	15.6	24.1	60.4
Richest	15.6	20.7	63.8	13.9	21.4	64.7
Parity	*P* < 0.000			*P* < 0.000		
1st Parity	19.0	20.4	60.6	15.9	22.8	61.3
2nd Parity	20.7	21.8	57.5	17.3	24.3	58.5
3rd Parity	26.5	23.9	49.6	24.8	26.4	48.9
More Parity	35.3	26.1	38.7	31.8	28.2	40.0
Any complication during delivery	*P* < 0.000			*P* < 0.000		
No	26.0	24.0	50.0	21.7	25.8	52.5
Yes	21.3	21.1	57.6	17.0	23.2	59.8
Number of ANC	*P* < 0.000			*P* < 0.000		
No visits	36.6	25.3	38.1	27.6	25.7	46.7
1–3 visits	30.4	24.6	45.0	28.5	27.3	44.2
4 & more visits	14.0	19.8	66.2	14.0	23.0	63.1
Received JSY Assistance	*P* < 0.000			*P* < 0.000		
No	18.1	20.7	61.3	19.3	24.5	56.2
Yes	27.6	23.8	48.7	17.3	22.1	60.6
%	**23.4**	**22.4**	**54.2**	**19.2**	**24.4**	**56.4**
N	**21,484**	**23,146**	**48,339**	**5588**	**6701**	**13,189**

Insufficient: < 24 h; Sufficient: 24-47 h; Extended: > 48 h; chi-square test (*p* < 0.000)

**Fig. 3:**
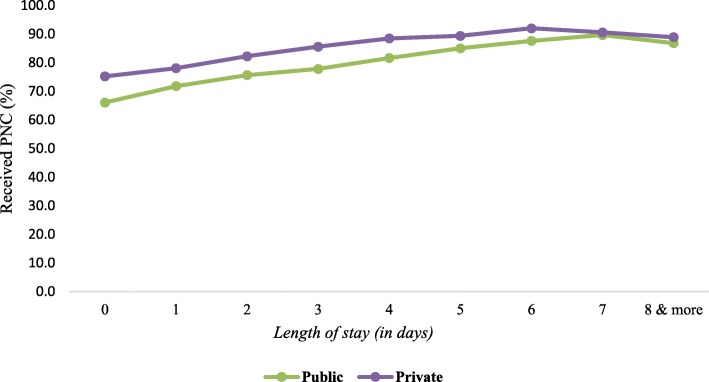
Women received postnatal care (PNC) at public and private health facilities

This paper also presents the percentage of women who receive postnatal care (PNC) after childbirth by their length of stay at public and private health facilities, as shown in Fig. 3. Results illustrate that women who stay longer at the facility utilize postnatal care more often than those who are discharged earlier.

The findings from the Cox regression show the effect of factors on length of stay after controlling other factors (Table 4). In contrast to the bivariate results, the risk of discharge at a given time does not vary much by women’s age as is evident from the hazard ratio (HR) which for age groups 25–29 and 30–39 is 0.982 and 0.973 in comparison to for the age group 15–24. There is a positive relationship between women’s education, household’s wealth, and women’s discharge rate at a given time. In other words, the length of stay has a linear association with the levels of education and, interestingly, an inverted U shape relationship with the wealth quintile of women. The increase in the length of stay occurs up to a certain level of wealth index, after which it reduces. The risk of early discharge is higher among rural women (HR-1.06) than the urban ones. Women belonging to lower caste groups (SC, ST, OBC) are more likely to stay for a longer duration (HR-0.919, 0.924, and 0.922 respectively) than those who belong to a higher caste.

**Table 4:** Associated factors of discharge of women from health facility: results from cox proportional hazard model, India, 2015–16

Variables		Hazard Ratio (C.I.)
Age	15–24 Years®	
	25–29 Years	0.982**(0.969–0.997)
	30–39 Years	0.973***(0.957–0.99)
	> 40 Years	0.985(0.944–1.028)
Education	Illiterate®	
	Primary	0.949***(0.93–0.969)
	Secondary	0.929***(0.913–0.945)
	Higher	0.923***(0.901–0.946)
Place of Residence	Urban®	
	Rural	1.06***(1.045–1.075)
Religion	Hindu®	
	Muslim	1.051***(1.033–1.069)
	Others	1.121***(1.098–1.143)
Caste	Others®	
	Schedule Castes	0.919***(0.902–0.936)
	Schedule Tribes	0.924***(0.906–0.943)
	Other Backward class	0.922***(0.908–0.936)
Wealth quintile	Poorest®	
	Poorer	0.957***(0.938–0.976)
	Middle	0.936***(0.917–0.955)
	Richer	0.958***(0.937–0.98)
	Richest	1.046***(1.02–1.072)
Parity	1st Parity®	
	2nd Parity	1.029***(1.015–1.043)
	3rd Parity	1.082***(1.062–1.103)
	More Parity	1.104***(1.079–1.131)
Any complications during delivery	No®	
	Yes	0.901***(0.891–0.911)
Number of ANC	No visits®	
	1–3 visits	1.023**(1.002–1.044)
	4 & more visits	0.918***(0.901–0.936)
Received JSY Assistance	No®	
	Yes	1.088***(1.073–1.103)
Mode of Delivery	Vaginal®	
	Cesarean	0.338***(0.333–0.344)
Type of facility	Public®	
	Private	1.029***(1.013–1.044)
	N	148,185

® refers Reference Category, *p* < 0.05 **, *p* < 0.01 ***

Women having higher parity stay for a shorter period. The hazard ratio of discharge among women with second, third, fourth and more parity is HR-1.0029, HR-1.082, and HR-1.104 respectively. The results further show that the early discharge rate is significantly lower among women who have any delivery complication (HR-0.901) and among those who have a cesarean delivery (HR-0.338). Antenatal care is the most important indicator of maternal health. Women who go for four or more ANC visits have a significantly lower risk of early discharge (HR-0.918). Women who receive the JSY assistance have a significantly higher discharge rate (HR-1.088) compared to the non-beneficiaries. Surprisingly, the findings suggest that after receiving the JSY assistance, women stay for a shorter time at the facility after childbirth.

The linkages between the length of stay after childbirth and the post-delivery complications are established by type of delivery, as presented in Table 5. Women staying at the hospital for one to 3 days have fewer post-delivery complications as compared to those who are discharged from the hospital on the same day itself. For instance, 12% of the women who stay for 3 days in the facility report high fever (within 2 months) after vaginal delivery as compared to 19% of such women who are discharged on the same day. Similarly, 20.7 vs 11.8% of women who stay for less than a day vs for 4 days, respectively, report high fever post a cesarean delivery. A higher percentage of women who stay in the hospital for more than 3 days’ report post-delivery fever. Post-delivery fever is observed more among women who deliver in public health facilities than among those who deliver in private facilities. Interestingly, there is no difference in the overall reporting of PPH by public and private health facility. The highest reporting of PPH is among women who stay for 4 days in public facilities and for eight or more days in private facilities. It is evident from this analysis that the reporting of PPH among those who stay for less than 4 days does not vary by length of stay and that afterwards, it increases with the length of stay. The highest proportion of PPH (25% or more) is among women who have a vaginal or public health delivery and stay for 4 days and among those who have a cesarean or private health delivery and stay for 8 or more days.

**Table 5:** Percentage of women who had post-delivery complications (massive vaginal bleeding (PPH) and very high fever) by their length of stay after childbirth at health facility, India, 2015–16

Duration of stay (in days)	Vaginal		Cesarean		Public		Private	
	PPH	Fever	PPH	Fever	PPH	Fever	PPH	Fever
0	19.3	18.8	27.2	20.7	20.3	19.7	18.5	16.4
1	18.6	14.8	22.3	16.1	19.1	15.5	17.9	13.2
2	19.0	13.2	26.2	20.9	19.6	14.1	19.0	12.2
3	18.7	12.1	19.3	13.9	18.4	12.5	19.8	11.5
4	24.8	13.5	22.1	11.8	26.6	14.8	21.0	10.9
5	21.4	12.6	20.5	12.2	22.5	13.1	19.9	11.9
6	21.0	15.1	18.4	13.3	19.4	14.3	18.9	13.4
7	21.4	14.2	20.9	13.7	21.1	14.0	20.9	13.6
8 & more	23.3	15.2	25.6	16.3	22.9	14.8	27.3	17.3
Total	19.3	14.6	21.9	14.4	19.9	15.2	20.0	13.3
**N**	**28,008**	**23,711**	**6341**	**4590**	**19,415**	**15,658**	**7999**	**5775**

## Discussion

Childbirth is considered a relatively safe event when it takes place at an institution or when the birth is attended to by a trained health professional. There is a substantial amount of maternal morbidity which can cause severe health complications and result in a prolonged length of stay after childbirth. We find that overall, a higher proportion of women who deliver in a public health facility are discharged within 2 days of the delivery as compared to those who deliver in a private health facility. Furthermore, our findings show a higher mean length of stay after delivery in the case of private health facilities than in the public health facilities. This finding concurs with the findings of earlier studies, including one multi-country study [12, 29]. A recent national level report stated that the Indian population relies more on private health providers for treatment [39] even though they are often profit-oriented. In private hospitals, the postnatal stay often occurs in a private room, which has an extra bed to accommodate the attendant family member. In contrast, in government hospitals, postnatal care is provided in open wards with many beds and no privacy. There is a scarcity of manpower in public health care facilities, which prevents them from rendering good services [﻿40﻿]. Some of the public health facilities don’t even have infrastructure that is up to the mark. Interestingly, for cesarean deliveries, women stay for longer in public than in private health facilities. One of the possible reasons for this pattern is that cesarean deliveries conducted in public facilities are genuinely complication-related and, therefore, necessitating that women stay for a longer period in order to recover fully. Women with vaginal deliveries stay for a shorter duration after childbirth than women with cesarean section deliveries, which validates the previous studies [12, 27].

Older women spend less time, particularly in public health facilities, after childbirth. However, in the multivariate analysis, women’s age was not found to have much effect on the length of stay. Women with higher parity stay for a shorter period irrespective of the mode and place of delivery. Higher parity women may be less educated and come from poor families and, therefore, they utilize less amount of healthcare services. Another possible reason could be that she wants to go home early because she has an older child at home. Our findings also suggest that Muslim women are more likely to take an early discharge and that they usually have higher parity as compared to women of other religions [41, 42]. It is common that women with higher parity have lower educational qualifications [43] and utilize lower quality services [44]. We found a positive relationship between length of stay and women’s education, households’ wealth, and urban residence. Interestingly, this relationship is only true for vaginal and not for cesarean deliveries. This could be credited to the fact that educated and economically-sound and urban women are well-informed and aware of the health risks involved in childbirth and that they have a high purchasing power to avail better quality services, which leads them to stay for longer after childbirth. Contrary to normal deliveries, we find that for cesarean deliveries, women with less education, those from poor households, those who are rural residents, and those from lower caste groups stay for a longer period. One of the possible reasons for this could be that these women go for a cesarean delivery only when there is a complication, while their counterparts go for it even when there is no complication. The long distance to the hospital from their residence in a rural area may be another reason that forces them to stay for longer in a hospital after having a cesarean delivery. Our findings suggest a need to further investigate the socio-economic disparities in the length of stay with more detailed information on what the reasons for early discharge are, who makes the decision relating to discharge, and what the delivery complications are. There is a need for qualitative research for explanations of these findings.

Women who are discharged with insufficient LOS after childbirth, whether in public or private hospitals, are mostly illiterate, live in rural areas, and belong to the poorest wealth quintile. The possible reason for this finding may be that illiterate women lack awareness about the importance of LOS, which is why they stay only for a limited number of days. On the other hand, in the case of poor households, it may not be affordable to stay for a sufficient number of days or the women could be working as day laborers and may need to get back to work.

Similar to prior research [25, 33], this paper also finds a positive effect of pregnancy complications on the postnatal length of stay. Policy interventions by the government too play a vital role in promoting a safe delivery approach as a significant increase in institutional deliveries has been observed after the implementation of JSY. The present paper also explores the effect of programmatic variables such as ANC visits and receipt of JSY on the length of stay. Women who go for four or more antenatal visits have a significantly longer stay. However, this study finds that women who receive the JSY assistance get an early discharge, particularly in public health facilities. An earlier study suggested that the scheme appeared to increase institutional deliveries among at-risk mothers as a significant increase was observed in maternal morbidities among women who had an institutional delivery after the implementation of JSY [7]. The scheme provides cash incentives to the frontline health workers to register pregnancies and bring women to deliver in health institutions. The finding of the present paper that women who receive JSY are less likely to stay for a longer time needs to be addressed in future research. The role of midwifery is crucial for delivering quality care and minimizing the number of days the women spend at the hospital. Yet in the Indian context, there is a huge shortage of health professionals at every level. Studies have shown that India lacks around 4 million midwives [45]. Retention of midwives, especially in rural areas, is a major challenge for many countries, including India [46].

Another key finding of this paper is that a longer length of stay may improve the utilization of PNC and reduce post-delivery complications, namely high fever and PPH. However, after a certain duration (around 3 days) of stay, post-delivery complications increase sharply with increase in the length of stay. This may be due to the fact that women who have complications during delivery may also have post-delivery complications and, therefore, stay for a longer time. In other words, a very long length of stay at a facility is attributed to poor health conditions, but a substantial length of stay is good for maternal health outcomes.

### Limitation

It is to be noted that the data used in this study does not include detailed information on post-delivery complications. The information on complications, that is, high fever and PPH, within 2 months of delivery during 5 year preceding the survey was sought. We are not sure whether the women had complications before or after discharge from the facility. In addition, we are not very clear about whether the women themselves sought discharge or whether it was the hospital that gave discharge.

A future study may be carried out to examine the relationship between the length of stay and health and wellbeing of Indian women and their infants and to explore the impact of professional midwifery care on these aspects. To get a better understanding, a hospital-based study may be carried out to gather more detailed information on LOS.

## Conclusion

This paper analyses a rarely discussed indicator, that is, length of stay in a health facility after childbirth. It was found that women who have a cesarean delivery in a public facility stay longer in comparison to those who deliver in private hospitals. Delivery cost is highly co-related with the length of stay in a private facility for both types of deliveries. We found that women with higher levels of education, with lower parity, those belonging to affluent households, and those who are urban residents stay in a facility for extended periods. The negative association of LOS with JSY receipt indicates that the JSY scheme has been successful to encourage women to come to the health institutions for delivery but not to make them aware about the importance of a sufficient LOS after childbirth. Finally, the paper reveals the key finding that women who are discharged on the same day receive lower levels of postnatal care and experience a higher proportion of post-delivery complications. We recommend that the government should focus on implementing the JSY scheme not only to elevate the levels of institutional birth but also to provide as much effective postnatal care as possible to keep women in the facility up to a necessary time period.

## Data Availability

We have provided details of the data in the methodology section. The NFHS-4 data can be obtained on request from the International Institute for Population Sciences, Mumbai, or DHS. The report and the survey tools are also available on the website: http://rchiips.org/nfhs/nfhs4.shtml

## Abbreviations

ANC: Antenatal Checkup
ASHA: Accredited Social Health Activists
C.I: Confidence Interval
DHS: Demographic and Health Surveys
HR: Hazards Ratio
JSY: *Janani Suraksha Yojana*
LOS: Length of Stay
MMR: Maternal Mortality Ratio
NFHS-4: National Family Health Survey (4th round)
NRHM: National Rural Health Mission
PNC: Post-natal Care
PPH: Postpartum Hemorrhage
WHO: World Health Organization
